# Predictors of In-Hospital Mortality among Patients with Pulmonary Tuberculosis: A Systematic Review and Meta-analysis

**DOI:** 10.1038/s41598-018-25409-5

**Published:** 2018-05-08

**Authors:** Carlos Podalirio Borges de Almeida, Patrícia Klarmann Ziegelmann, Rachel Couban, Li Wang, Jason Walter Busse, Denise Rossato Silva

**Affiliations:** 1 Respiratory Sciences Program, Universidade Federal do Rio Grande do Sul, Porto Alegre, Rio Grande do Sul Brazil; 2 Statistics Department and Postgraduate Program in Epidemiology, Universidade Federal do Rio Grande do Sul, Porto Alegre, Rio Grande do Sul Brazil; 3 The Michael G. DeGroote Institute for Pain Research and Care, McMaster University, Hamilton, Ontario Canada; 4 The Michael G. DeGroote Institute for Pain Research and Care, McMaster University, Hamilton, Ontario Canada; 5 Departments of Anesthesia and Health Research Methods, Evidence and Impact, and The Michael G. DeGroote Institute for Pain Research and Care, McMaster University, Hamilton, Ontario Canada; 6 Pulmonology Division, Faculty of Medicine, Universidade Federal do Rio Grande do Sul; Hospital de Clínicas de Porto Alegre, Porto Alegre, Rio Grande do Sul Brazil; 7 Respiratory Sciences Program, Universidade Federal do Rio Grande do Sul, Porto Alegre, Brazil

## Abstract

**Background:** There is uncertainty regarding which factors are associated with in-hospital mortality among patients with pulmonary TB (PTB). The aim of this systematic review and meta-analysis is to identify predictors of in-hospital mortality among patients with PTB. **Methods:** We searched MEDLINE, EMBASE, and Global Health, for cohort and case-control studies that reported risk factors for in-hospital mortality in PTB. We pooled all factors that were assessed for an association, and presented relative associations as pooled odds ratios (ORs). **Results:** We identified 2,969 records, of which we retrieved 51 in full text; 11 cohort studies that evaluated 5,468 patients proved eligible. Moderate quality evidence suggested an association with co-morbid malignancy and in-hospital mortality (OR 1.85; 95% CI 1.01–3.40). Low quality evidence showed no association with positive sputum smear (OR 0.99; 95% CI 0.40–2.48), or male sex (OR 1.09, 95% CI 0.84–1.41), and very low quality evidence showed no association with diabetes mellitus (OR 1.31, 95% IC 0.38–4.46), and previous TB infection (OR 2.66, 95% CI 0.48–14.87). **Conclusion:** Co-morbid malignancy was associated with increased risk of in-hospital death among pulmonary TB patients. There is insufficient evidence to confirm positive sputum smear, male sex, diabetes mellitus, and previous TB infection as predictors of in-hospital mortality in TB patients.

## Introduction

Tuberculosis (TB) continues to be a major public health issue worldwide, particularly in low and middle-income countries despite rigorous efforts to contain its spread and implementation of effective treatment strategies. In 2014 an estimated 12 million people worldwide were living with active pulmonary TB, with 9.6 million new cases and 1.5 million deaths due to TB occurring annually^1–7^.

TB does not usually require hospital admission for treatment, but if symptoms such as shortness of breath, and deterioration in a systemic condition are present, hospitalization may be necessary. A large proportion of patients with TB are hospitalized^8,9^, and estimates of in-hospital mortality range from 2% to 12%^10–14^; most of the current costs of TB treatment result from hospitalization^15^.

A variety of predictors have been associated with a greater risk of death among TB patients, including poverty, homelessness, alcohol or drug addiction, irregular or inadequate treatment, late diagnosis of the disease, multidrug-resistant TB (MDR-TB), and advanced age^4,6^. Human immunodeficiency virus (HIV) infection is an important factor related to the increased morbidity and mortality of TB in different world regions^4,10^. In addition, diabetes has been reported to be associated with increased risk of mortality^16–18^. Also, men have higher rates of mortality and worse outcomes compared with women^19,20^. Previous TB with multiple treatments has also been associated with in-hospital mortality^21–23^. Furthermore, patients with malignant tumors are immunocompromised and can have unusual clinical presentations, both related to delayed diagnosis and high mortality^24–26^.

In TB program monitoring, TB deaths are crucial indicators of the impact of TB control measures^10–14^, especially in areas with high HIV and TB prevalence. Data on TB deaths should provide us with a better understanding of the factors associated with these deaths and help guide interventions to reduce mortality; however, there is uncertainty regarding which factors are associated with in-hospital mortality among patients with pulmonary TB^10^.

We therefore conducted a systematic review and meta-analysis to establish predictors of in-hospital mortality among patients with pulmonary TB.

## Methods

### Search strategy

We used a multimodal search strategy focused on 3 bibliographical databases (MEDLINE, EMBASE and Global Health). An experienced librarian (RC) used medical subject headings, adding terms and keywords from a preliminary search to develop the database search strategies. In each database, the librarian used an iterative process to refine the search strategy through testing several search terms and incorporating new search terms as new relevant citations were identified. There were no language restrictions. The search included the following databases from inception to November 2015: MEDLINE, EMBASE and Global Health. The search consisted of three concepts combined using the AND operator^1^: tuberculosis^2^, hospitalization and^3^ mortality (Appendix 1). The protocol of this study was published elsewhere^27^.

### Study selection

#### Eligibility criteria

Eligible trials met the following criteria^1^: cohort or case-control design^2^; explored risk factors for in-hospital mortality among patients with pulmonary TB in an adjusted analysis.

#### Assessment of study eligibility

Two reviewers (CPBA and DRS) trained in health research methodology screened, independently and in duplicate, the titles and abstracts of all citations identified in our search. The same reviewers screened all full text articles for eligibility; disagreements were resolved by consensus, with consultation of a third investigator (JWB) when resolution could not be achieved. We measured agreement between reviewers with the kappa statistic to assess the reliability of full-text review using the guidelines proposed by Landis and Koch^28^: <0.20 as slight agreement, 0.21–0.40 as fair agreement, 0.41–0.60 as moderate agreement, 0.61–0.80 as substantial agreement and >0.80 as almost perfect agreement.

#### Assessment of study quality

Two reviewers (CPBA and DRS) assessed risk of bias for each eligible study, independently and in duplicate, using the Newcastle-Ottawa quality assessment scale (NOS) for Cohort Studies^29^. The scale consists of nine items that cover three dimensions^1^: patient selection (4 items)^2^; comparability of cohorts on the basis of the design or analysis (2 items); and^3^ assessment of outcome (3 items). A point is awarded for each item that is satisfied by the study. The total score therefore ranges from zero to nine, with higher scores indicating higher quality. A total score ≥7 represents high quality.

#### Data Extraction and Analysis

Two reviewers (CPBA and DRS) extracted data from each eligible study, including demographic information (e.g. sex, age, race), methodology, and all reported predictors.

We performed meta-analysis for all predictors that were reported by more than one study. We used odds ratios (ORs) with associated 95% CI to measure the association of binary predictors and in-hospital mortality. We used random effects models for all meta-analyses. If a study reported more than 1 regression model, we used data from the most fully adjusted model presented. We also presented the results from the predictors explored by the studies but that were not eligible for meta-analysis.

We evaluated heterogeneity for all pooled estimates through visual inspection of forest plots, because statistical tests of heterogeneity can be misleading when sample sizes are large and CIs are therefore narrow^30^. We used the software R.

#### Publication bias

For meta-analyses with at least 10 studies, we assessed publication bias by visual assessment of asymmetry of the funnel plot and performed the Begg rank correlation test^31^.

#### Quality of evidence

We used the Grading of Recommendations Assessment, Development and Evaluation (GRADE) approach to summarize the quality of evidence for all meta-analyses^32^. We categorized the confidence in estimates (quality of evidence) as high, moderate, low or very low, on the basis of risk of bias^33^, imprecision^34^, indirectness, inconsistency^35^ and publication bias^36^. We used GRADE evidence profiles to provide a succinct, easily digestible presentation of the quality of evidence and magnitude of associations^32^. In case of doubt or missing details about the studies, authors were contacted for clarification.

#### Ethics and Dissemination

This study is based on published data, and therefore ethical approval was not a requirement. This systematic review and meta-analysis is expected to serve as a basis for evidence to reduce in-hospital mortality in TB patients, and as a guide for future research based on identified knowledge gaps. It is anticipated that findings from this review will be useful for informing policy, practice and research priorities, improving the management of in-hospital TB patients. We also plan to update the review in the future to monitor changes and guide health services and policy solutions.

## Results

### Search Results and Study Characteristics

**Figure 1:**
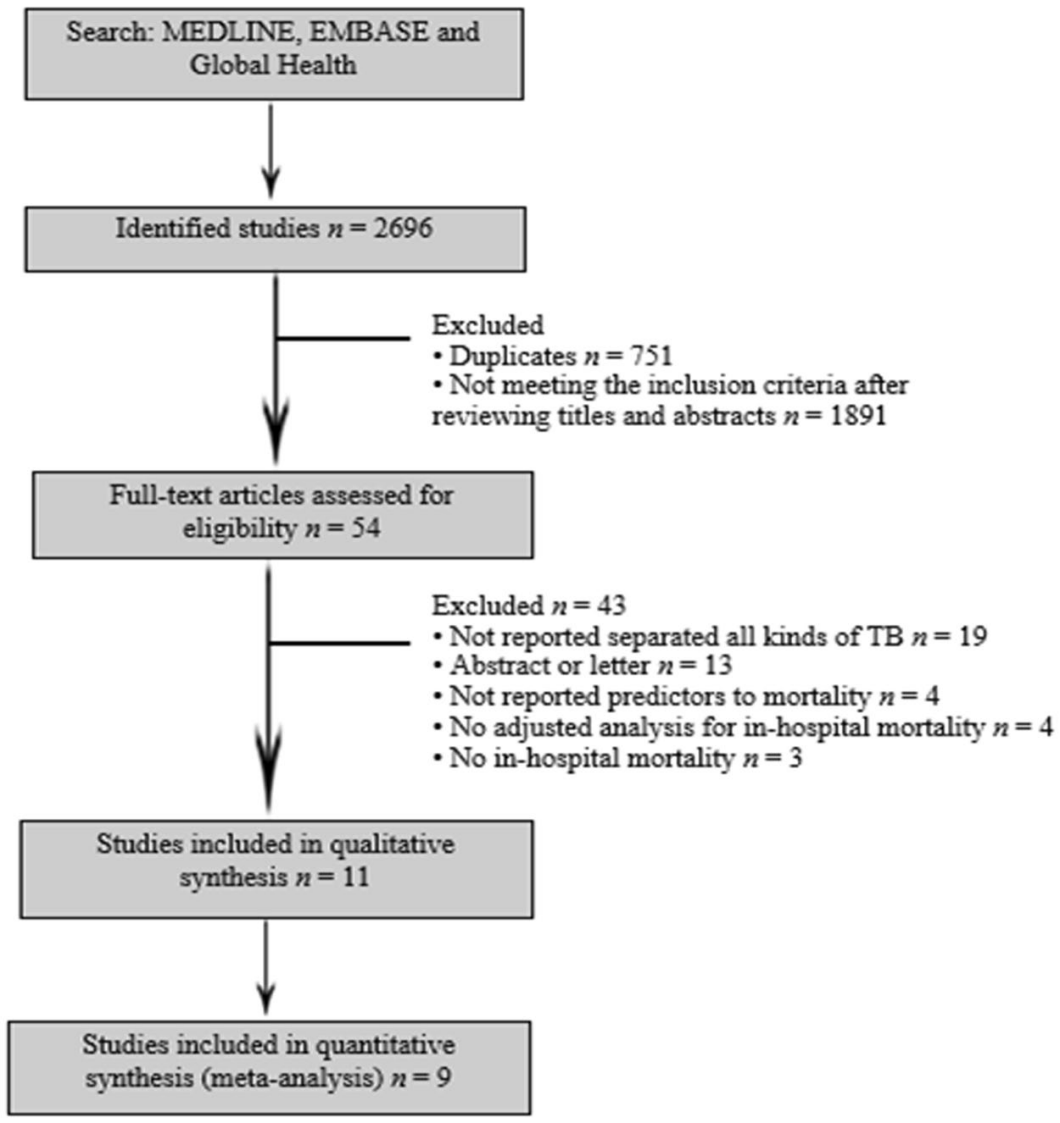
Flow diagram of study selection.

We identified 2,969 unique records, of which we retrieved 51 English and 3 non-English language articles in full text; 11 cohort studies, published between 2003 and 2013, that evaluated 5,468 patients proved eligible. Figure 1 shows the study selection flow diagram. There was substantial agreement (κ = 0.64) at the titles and abstract screening stage and perfect agreement (κ = 1.00) between reviewers at the full-text review stage.

All 11 eligible studies^1,4,15,37–44^ were single-center and there was one non-English (Chinese) study included in our analysis. Two studies^38,42^ were conducted in Japan, two^40,41^ in Taiwan, three^15,39,43^ in Korea, one^37^ in Germany, one^4^ in Israel, one^1^ in Iran and one^44^ in China. One study^39^ used TB-related mortality as defined by the World Health Organization (the number of TB patients who died during treatment, irrespective of cause)^45^, two^38,42^ used all-cause mortality, and eight^1,4,15,37,40,41,43,44^ used TB-related mortality as judged by the investigators. The majority (9 of 11)^1,4,15,37,39–41,43,44^ acquired data from medical records, with eight retrospective cohorts^1,4,37–42^ and one prospective cohort study^15^ (Table 1).

**Table 1:** Studies describing in-hospital mortality among pulmonary tuberculosis patients.

First author, reference	Year of publication	Definition of TB death*	Country	Sample size	No. Deaths (%)	Predictors
Alavi-Naini^1^	2013	Investigators judgment of TB death	Iran	715	75 (13.9%)	Smoking, hepatites, DM, Hx of previous TB, anemia, drug abuse, positive sputum smear
Erbes^37^	2006	Investigators judgment of TB death	Germany	58	15 (25.9%)	Acute renal failure, mechanical ventilation, pneumonia, chronic pancreatitis, sepsis, ARDS
Horita^38^	2012	All-cause mortality	Japan	244	48 (19.7%)	Age, oxygen requirement, albumin, ADL
Kim^15^	2010	Investigators judgment of TB death	Korea	156	21 (13.5%)	Male sex, old age, underprivileged, predisposing factors, AFB smear, CRP, lung involvement, high NRS
Kim^39^	2012	WHO definition	Korea	269	82 (30.5%)	Admission Route, AFB Smear Positivity, albumin, BUN, creatinine, CRP, Drug-resistance TB, general weakness, Hb, hx of stopping anti-TB medication, hospital length of stay, initial ICU care, lymphocyte, poor oral intake, severity on chest X-ray, sodium, total cholesterol, under treatment for TB, WBC
Lee^40^	2003	Investigators judgment of TB death	Taiwan	41	27 (64.8%)	Multiple organ failure, consolidation on chest X-ray
Lin^41^	2009	Investigators judgment of TB death	Taiwan	59	40 (67.8%)	Acute renal failure, gastrointestinal bleeding, multi-organ dysfunction syndrome, nosocomial pneumonia, treatment delay > 30 days
Lubart^4^	2007	Investigators judgment of TB death	Israel	461	65 (14%)	Older age, IHD, cachexia, corticosteroid use, low albumin level
Okamura^42^	2013	All-cause mortality	Japan	246	27 (11%)	Serum Albumin, total lymphocite – cat 1, total limphocite – cat2, total limphocite – cat3
Ryu^43^	2006	Investigators judgment of TB death	Korea	32	16 (50%)	APACHE II, sepsis, tuberculous-destroyed lungs
Sun^44^	2011	Investigators judgment of TB death	China	62	36 (58%)	APACHE II, liver damage, respiratory failure, fungal infection

ADL = activities of daily living; APACHE II = Acute Physiology and Chronic Health Evaluation; ARDS = acute respiratory distress syndrome; DM = Diabetes Mellitus; Hx = history; IHD = ischemic heart disease; TB = tuberculosis; PTB = pulmonary TB; NR = Not reported; WHO = World Health Organization.

### Risk of bias

Overall, the quality, evaluated by the NOS checklist for the outcome “mortality”, was high (Table 2). We did not have a sufficient number of studies in our meta-analyses to assess publication bias.

**Table 2:** Newcastle-Ottawa scoring system for cohort studies.

Study	Selection score	Comparability score	Outcome score	Total score
Alavi-Naini^1^	2	2	3	7
Erbes^37^	2	2	3	7
Horita^38^	2	2	3	7
Kim (2010)^15^	2	2	3	7
Kim (2012)^39^	2	2	3	7
Lee^40^	2	2	3	7
Lin^41^	2	2	3	7
Lubart^4^	2	2	3	7
Okamura^42^	2	2	3	7
Ryu^43^	2	2	3	7
Sun^44^	2	2	3	7

### Predictors of in-hospital mortality

A total of 11 studies, involving a total of 2343 patients, reported the association of 60 factors with in-hospital mortality^1,4,15,37–42^. On the basis of our criteria, we conducted meta-analyses for 5 predictors of in-hospital mortality^1^: acid-fast bacilli (AFB) smear positive^2^, diabetes mellitus^3^, malignancy^4^, history of previous TB, and^5^ male sex.

**Figure 2:**
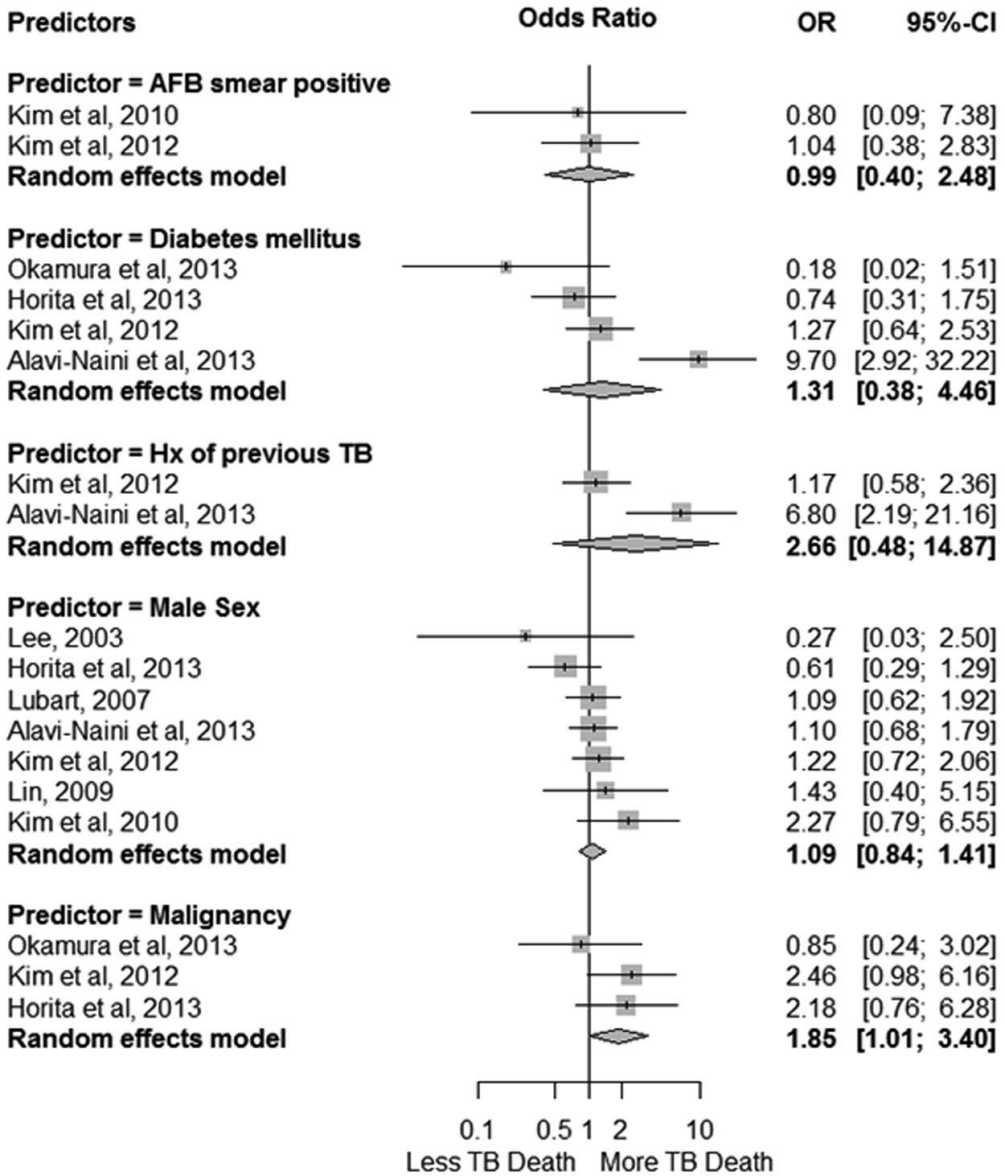
Association between AFB smear positive, Diabetes Mellitus, Hx of previous TB, Malignancy, male sex and in-hospital mortality among pulmonary TB patients.

Moderate quality evidence showed a significant association between malignancy and in-hospital mortality among TB patients (OR 1.85; 95% CI 1.01–3.40). Low quality evidence showed no association between in-hospital mortality and AFB smear positive test (OR 0.99; 95% CI 0.40–2.48), or male sex (OR 1.09; 95% CI 0.84–1.41). Very low quality evidence showed no association between mortality and diabetes mellitus (OR 1.31; 95% CI 0.38–4.46), or previous TB (OR 2.66; 95% CI: 0.48–14.87) (Fig. 2; Table 3).

**Table 3:** GRADE Evidence Profile: Predictors of in-hospital mortality among TB patients.

Predictor/Time/N° of patients	N° of studies	Risk of bias	Inconsistancy	Indirectness	Imprecision	Quality	Relative effect (95% CI)
AFB smear positive/At baseline/1116 patients	2	No serious risk of bias	No serious inconsistancy	No serious indirectness	Serious imprecision	Low	OR 0.99 (0.40–2.48)
DM/At baseline/2165 patients	4	No serious risk of bias	Serious inconsistancy	No serious indirectness	Serious imprecision	Very low	OR 1.31 (0.38–4.46)
Hx of previous TB/At baseline/1675 patients	2	No serious risk of bias	Serious inconsistancy	No serious indirectness	Serious imprecision	Very low	OR 2.66 (0.48–14.87)
Male sex/At baseline/1880 patients	7	No serious risk of bias	No serious inconsistancy	No serious indirectness	Serious imprecision	Low	OR 1.09 (0.84–1.41)
Malignancy/At baseline/694 patients	3	No serious risk of bias	No serious inconsistancy	No serious indirectness	No serious imprecision	Moderate	OR 1.85 (1.01–3.40)

*DM = Diabetes Mellitus; Hx = history.

Table 4 presents the associations with in-hospital mortality for the factors that were not amenable to meta-analysis.

**Table 4:** Unpooled predictors for in-hospital mortality among TB patients.

Predictors	OR/HR*	p-value
**Sociodemographic factors**		
Age (for 1 year increase)	—	0.007
Old age (>65 years)	5.7 (0.8–38.9)	0.076
Older age	—	<0.001
Underprivileged	4.1 (0.8–21.4)	0.098
**Substance use**		
Drug abusers	7.8 (2.4–25.5)	0.008
Smoking	12.9 (3.9–27.3)	0.001
**Previous TB**		
Tuberculous-destroyed lungs	6.61 (1.21–36.04)*	0.029
History of stopping anti-TB medication	4.58 (0.90–23.38)	0.068
**Symptoms**		
General weakness	1.23 (0.35–4.32)	0.744
Cachexia	—	<0.001
**Chest X-ray**		
Consolidation	7.73 (1.03–57.68)*	0.046
Extensive radiographic lung involvement	5.0 (0.6–42.8)	0.140
Severity on chest X-ray - Mild	1.00	0.796
Severity on chest X-ray -Moderate	1.63 (0.34–7.83)	0.543
Severity on chest X-ray - Severe	1.37 (0.26–7.16)	0.706
**Laboratorial exams**		
Sputum AFB smear >3	2.00 (0.59–6.75)*	0.264
Multidrug-resistant tuberculosis	2.65 (0.28–25.33)*	0.397
Drug-resistance TB	2.06 (0.69–6.11)	0.195
Hb	1.20 (0.40–1.60)	0.742
Lymphocyte	1.99 (0.79–4.97)	0.143
WBC	2.06 (0.89–4.78)	0.091
Total Lymphocite – cat 1	1.00	—
Total Limphocite – cat 2	0.13 (0.03–0.59)	0.010
Total Limphocite – cat 3	0.46 (0.13–1.65)	0.235
Albumin	1.76 (0.68–4.53)	0.245
Serum Albumin	0.15 (0.06–0.37)	<0.0001
Albumin (for 1 g/dl increase)	0.22 (–)	0.003
Low albumin level	—	<0.001
CRP, g/L	1.00 (0.87–1.15)	0.883
CRP, mg/dL	1.62 (0.38–6.95)	0.517
BUN	3.23 (1.23–8.49)	0.018
Creatinine	2.00 (0.60–6.64)	0.256
Sodium	2.48 (0.99–6.21)	0.052
Total cholesterol	0.87 (0.18–4.11)	0.857
**Findings during hospitalization**		
Admission Route	0.83 (0.33–2.08)	0.695
Initial admission ward - ICU	6.17 (2.08–18.32)	0.001
Under treatment for PTB at admission	3.35 (1.12–9.99)	0.030
APACHE II score	4.91 (1.99–12,11)*	<0.01
APACHE II score >20	4.90 (1.43–16.80)*	0.012
Treatment delay >30 days	2.37 (0.49–69.4)	—
Nosocomial pneumonia	5.77 (1.33–44.36)	—
Nosocomial pneumonia	—	0.002
Respiratory failure	4.03 (1.56–10.38)*	<0.01
Drug hepatitis	12.3 (6.7–24.7)	0.001
Liver damage	3.96 (1.23–12.1)*	<0.05
Gastrointestinal bleeding	0.5 (0.203–26.18)	—
Acute renal failure	0.6 (0.215–7.15)	—
Acute renal failure	—	0.001
Fungal infection	3.44 (1.23–9.62)*	<0.05
Multiple organ failure	0.60 (0.14–2.60)*	0.495
Multi-organ dysfunction syndrome	8.59 (1.85–101.27)	—
Multiple organ failure	2.65 (1.16–6.04)*	0.020
Sepsis	5.84 (1.63–20.95)*	0.007
Sepsis	—	0.001
Hospital length of stay	1.51 (0.58–3.91)	0.395
Anemia	19.8 (5.6–35.5)	<0.0001
Oxygen requirement	2.29 (−)	0.132
Mechanical ventilation	—	0.002
Chronic pancreatitis	—	0.001
ARDS	—	0.008
IHD	—	<0.001
**Other**		
Poor oral intake	0.94 (0.24–3.71)	0.930
Activity of Daily living (for 1 point increase)	0.58 (−)	0.141
High NRS	23.5 (2.9–194.2)	0.003
Predisposing factors	9.1 (1.5–56.8)	0.019
Corticosteroid use	—	<0.001

## Discussion

We found moderate quality evidence that co-morbid malignancy was associated with increased in-hospital mortality among TB patients. Low quality evidence showed that sex and AFB smear positive were not associated with in-hospital mortality, and very low quality evidence showed no association with previous TB infection and diabetes mellitus.

Our review has a number of strengths. Our search, which had no language restrictions, was designed and implemented by a research librarian, and literature screening and data extraction were performed independently and in duplicate by two reviewers using pretested, standardized extraction forms. The main limitation of our review was the small numbers of events that contributed to our meta-analyses, resulting in wide estimates of precision for our pooled measures of association.

Other studies^24–26^ also found that malignancy increases the risk of death in TB patients. Patients with malignant tumors are immunocompromised due to the local or systemic effects of the disease itself, as well as to the treatment regimens, which can impair the immune system and make these patients particularly susceptible to developing TB^46^. In addition, TB can have an unusual clinical presentation, making diagnosis more difficult in these patients, contributing to delay in diagnosis and high mortality rates^47,48^.

While not significantly associated with mortality in our review, previous TB has been reported to be associated with in-hospital mortality in many studies^1,21–23^. Patients who undergo multiple treatment regimens for TB can develop resistance to drugs with the subsequent emergence of MDR-TB and XDR-TB, conditions highly associated with greater risk of death^21^. Further, in settings other than hospitals, studies^49,50^ have demonstrated that smear positive patients have a better prognosis regarding mortality than smear negative patients. Indeed, indicators of atypical manifestations, such as smear-negative sputum, were associated with delayed diagnosis and mortality^12,51^. Recently, a retrospective cohort study from Brazil^6^ reported a high mortality rate during hospitalization (16.1%), and negative sputum smear microscopy was an in-hospital mortality predictor in the population studied. However, patients with pulmonary and extrapulmonary TB were included in this study.

We did not find a significant association between male sex and in-hospital mortality among pulmonary TB patients. Worldwide TB notification data show that far more men than women have TB^7^. Some studies showed that mortality rates are higher in females during their reproductive years, but after that they are higher in men^19,20^.

Diabetes was also not associated with mortality in pulmonary TB patients in this study. Only one study^1^ included in this meta-analysis showed that diabetes was a predictor of mortality in TB patients, possibly because they included a larger number of diabetes patients (18% of the enrolled individuals). Some studies^1,16–18^ have found that diabetes increases risk of early mortality during TB treatment. This effect may be explained by impaired TB treatment response^16^.

In conclusion, the presence of malignancy was significantly associated with in-hospital death in pulmonary TB patients. Other predictors were not associated with in-hospital mortality in TB patients, probably due to the small number of events. Further research should explore promising predictors of in-hospital mortality in large prospective studies.

## Electronic supplementary material

Supplementary Information

## References

[1] Alavi-Naini R, Moghtaderi A, Metanat M, Mohammadi M, Zabetian M (2013). Factors associated with mortality in tuberculosis patients. J Res Med Sci.

[2] Haque G (2014). Prognostic factors in tuberculosis related mortalities in hospitalized patients. Tuberc Res Treat.

[3] Lawn SD, Zumla AI (2011). Tuberculosis. Lancet.

[4] Lubart E, Lidgi M, Leibovitz A, Rabinovitz C, Segal R (2007). Mortality of patients hospitalized for active tuberculosis in Israel. Isr Med Assoc J.

[5] Lui G (2014). High mortality in adults hospitalized for active tuberculosis in a low HIV prevalence setting. PLoS One.

[6] Silva DR, Menegotto DM, Schulz LF, Gazzana MB, Dalcin PT (2010). Factors associated with mortality in hospitalized patients with newly diagnosed tuberculosis. Lung.

[7] WHO. Global Tuberculosis Report 2015. Geneva: World Health Organization. Available at: www.who.int (2015).

[8] Silva DR, Silva LP, Dalcin PT (2014). Tuberculosis in hospitalized patients: clinical characteristics of patients receiving treatment within the first 24 h after admission. J Bras Pneumol.

[9] Sreeramareddy CT, Panduru KV, Menten J, Van, den Ende J (2009). Time delays in diagnosis of pulmonary tuberculosis: a systematic review of literature. BMC Infect Dis.

[10] Hansel NN, Merriman B, Haponik EF, Diette GB (2004). Hospitalizations for tuberculosis in the United States in 2000: predictors of in-hospital mortality. Chest.

[11] Hansel NN, Wu AW, Chang B, Diette GB (2004). Quality of life in tuberculosis: patient and provider perspectives. Qual Life Res.

[12] Rao VK, Iademarco EP, Fraser VJ, Kollef MH (1998). The impact of comorbidity on mortality following in-hospital diagnosis of tuberculosis. Chest.

[13] Singleton L (1997). Long-term hospitalization for tuberculosis control. Experience with a medical-psychosocial inpatient unit. JAMA.

[14] Greenaway C (2002). Delay in diagnosis among hospitalized patients with active tuberculosis–predictors and outcomes. Am J Respir Crit Care Med.

[15] Kim HJ (2010). The impact of nutritional deficit on mortality of in-patients with pulmonary tuberculosis. Int J Tuberc Lung Dis.

[16] Faurholt-Jepsen D (2013). Diabetes is a strong predictor of mortality during tuberculosis treatment: a prospective cohort study among tuberculosis patients from Mwanza, Tanzania. Trop Med Int Health.

[17] Reed GW (2013). Impact of diabetes and smoking on mortality in tuberculosis. PLoS One.

[18] Workneh MH, Bjune GA, Yimer SA (2016). Diabetes mellitus is associated with increased mortality during tuberculosis treatment: a prospective cohort study among tuberculosis patients in South-Eastern Amahra Region, Ethiopia. Infect Dis Poverty.

[19] Holmes CB, Hausler H, Nunn P (1998). A review of sex differences in the epidemiology of tuberculosis. Int J Tuberc Lung Dis.

[20] Weiss MG, Sommerfeld J, Uplekar MW (2008). Social and cultural dimensions of gender and tuberculosis. Int J Tuberc Lung Dis.

[21] Chung-Delgado K, Guillen-Bravo S, Revilla-Montag A, Bernabe-Ortiz A (2015). Mortality among MDR-TB cases: comparison with drug-susceptible tuberculosis and associated factors. PLoS One.

[22] de Faria Gomes NM (2015). Differences between Risk Factors Associated with Tuberculosis Treatment Abandonment and Mortality. Pulm Med.

[23] Pepper DJ, Schomaker M, Wilkinson RJ, de AV, Maartens G (2015). Independent predictors of tuberculosis mortality in a high HIV prevalence setting: a retrospective cohort study. AIDS Res Ther.

[24] Feng JY (2011). Initial presentations predict mortality in pulmonary tuberculosis patients–a prospective observational study. PLoS One.

[25] Lin CH (2014). Tuberculosis mortality: patient characteristics and causes. BMC Infect Dis.

[26] Pina JM (2006). [Excess mortality due to tuberculosis and factors associated to death in and annual cohort of patients diagnosed of tuberculosis]. Rev Clin Esp.

[27] Almeida CP (2016). Predictors of in-hospital mortality among patients with pulmonary tuberculosis: a protocol of systematic review and meta-analysis of observational studies. BMJ Open.

[28] Landis JR, Koch GG (1977). The measurement of observer agreement for categorical data. Biometrics.

[29] Wells G. A. *et al*. The Newcastle-Ottawa Scale (NOS) for assessing the quality of non-randomised studies in meta-analyses. Ottawa Hospital Research Institute. Available at: http://www.ohri.ca/programs/clinical_epidemiology/oxford.asp (2016).

[30] Rucker G, Schwarzer G, Carpenter JR, Schumacher M (2008). Undue reliance on I(2) in assessing heterogeneity may mislead. BMC Med Res Methodol.

[31] Begg CB, Mazumdar M (1994). Operating characteristics of a rank correlation test for publication bias. Biometrics.

[32] Atkins D (2004). Grading quality of evidence and strength of recommendations. BMJ.

[33] Guyatt GH (2011). GRADE guidelines: 4. Rating the quality of evidence–study limitations (risk of bias). J Clin Epidemiol.

[34] Guyatt GH (2011). GRADE guidelines 6. Rating the quality of evidence–imprecision. J Clin Epidemiol.

[35] Guyatt GH (2011). GRADE guidelines: 7. Rating the quality of evidence–inconsistency. J Clin Epidemiol.

[36] Guyatt GH (2011). GRADE guidelines: 5. Rating the quality of evidence–publication bias. J Clin Epidemiol.

[37] Erbes R (2006). Characteristics and outcome of patients with active pulmonary tuberculosis requiring intensive care. Eur Respir J.

[38] Horita N (2013). Development and validation of a tuberculosis prognostic score for smear-positive in-patients in Japan. Int J Tuberc Lung Dis.

[39] Kim CW (2012). Risk factors related with mortality in patient with pulmonary tuberculosis. Tuberc Respir Dis (Seoul).

[40] Lee PL (2003). Patient mortality of active pulmonary tuberculosis requiring mechanical ventilation. Eur Respir J.

[41] Lin SM (2009). Predictive factors for mortality among non-HIV-infected patients with pulmonary tuberculosis and respiratory failure. Int J Tuberc Lung Dis.

[42] Okamura K (2013). Hypoalbuminemia and lymphocytopenia are predictive risk factors for in-hospital mortality in patients with tuberculosis. Intern Med.

[43] Ryu YJ (2007). Prognostic factors in pulmonary tuberculosis requiring mechanical ventilation for acute respiratory failure. Respirology.

[44] Sun J, Fang K, Ren DH, Sheng XL (2011). [A study of the prognostic factors associated with mortality in critically ill patients with tuberculous]. Zhonghua Jie He He Hu Xi Za Zhi.

[45] Who. World Health Organization. Global Tuberculosis Programme. A framework for effective tuberculosis control. WHO/TB/94. 179. Geneva, Switzerland. Available at: www.who.int (1994).

[46] Kamboj M, Sepkowitz KA (2006). The risk of tuberculosis in patients with cancer. Clin Infect Dis.

[47] Kim HR (2008). Solid-organ malignancy as a risk factor for tuberculosis. Respirology.

[48] Silva FA, Matos JO, de QMF, Nucci M (2005). Risk factors for and attributable mortality from tuberculosis in patients with hematologic malignances. Haematologica.

[49] Balabanova Y (2006). The Directly Observed Therapy Short-Course (DOTS) strategy in Samara Oblast, Russian Federation. Respir Res.

[50] Dewan PK (2004). Risk factors for death during tuberculosis treatment in Orel, Russia. Int J Tuberc Lung Dis.

[51] Naalsund A, Heldal E, Johansen B, Kongerud J, Boe J (1994). Deaths from pulmonary tuberculosis in a low-incidence country. J Intern Med.

