# Supplementary material for: Predictors of In-Hospital Mortality among Patients with Pulmonary Tuberculosis: A Systematic Review and Meta-analysis
Source: Sci Rep. 2018 May 8;8:7230. doi: 10.1038/s41598-018-25409-5 (PMC5940698; doi:10.1038/s41598-018-25409-5)
Supplement: Supplementary file 1 — Supplementary Information [file 41598_2018_25409_MOESM1_ESM.doc]

**Title:** Predictors of In-Hospital Mortality among Patients with Pulmonary Tuberculosis: A Systematic Review and Meta-analysis.

**Authors:** Carlos Podalirio Borges de Almeida, DC, MSc, Patrícia Klarmann Ziegelmann, PhD, Rachel Couban, MA, MISt, Li Wang, PhD, Jason Walter Busse, DC, PhD, Denise Rossato Silva, MD, PhD

**Appendix 1: Search strategies for MEDLINE, EMBASE and Global Health**

Database: Ovid MEDLINE(R) In-Process & Other Non-Indexed Citations, Ovid MEDLINE(R) Daily and Ovid MEDLINE(R) <1946 to Present>

Search Strategy:

--------------------------------------------------------------------------------

1 tuberculosis/ or exp tuberculosis, pulmonary/ (120663)

2 tuberc*.mp. (232310)

3 1 or 2 (232668)

4 exp Hospitalization/ (168145)

5 in-hospital.mp. (56173)

6 hospitalization.mp. (137044)

7 hospitalized.mp. (72827)

8 or/4-7 (306697)

9 3 and 8 (3597)

10 mortality/ or hospital mortality/ (60707)

11 mortalit*.mp. (537083)

12 10 or 11 (537083)

13 9 and 12 (585)

14 "Treatment outcome of new smear positive pulmonary tuberculosis patients in Penang, Malaysia".fc_titl. (1)

15 "Predictive factors for mortality among non-HIV-infected patients with pulmonary tuberculosis and respiratory failure".fc_titl. (1)

16 "Hospitalizations for Tuberculosis in the United States in 2000".fc_titl. (1)

17 "Initial presentations predict mortality in pulmonary tuberculosis patients".fc_titl. (1)

18 "Risk factors related with mortality in patient with pulmonary tuberculosis".fc_titl. (1)

19 "Factors associated with mortality in hospitalized patients with newly diagnosed tuberculosis".fc_titl. (1)

20 "High mortality in adults hospitalized for active tuberculosis in a low HIV prevalence setting".fc_titl. (1)

21 "Mortality of Patients Hospitalized for Active Tuberculosis in Israel".fc_titl. (1)

22 "Prognostic factors in tuberculosis related mortalities in hospitalized patients".fc_titl. (1)

23 "Factors associated with mortality in tuberculosis patients".fc_titl. (1)

24 or/14-23 (10)

25 13 and 24 (8)

26 24 not 25 (2)

EMBASE

Database: Embase <1974 to 2014 November 25>

Search Strategy:

--------------------------------------------------------------------------------

1 tuberculosis/ or lung tuberculosis/ (158425)

2 tubercul*.mp. (259935)

3 1 or 2 (259935)

4 hospitalization/ or hospitalization.mp. (268589)

5 hospitalized.mp. (93919)

6 in-hospital.mp. (80244)

7 or/4-6 (390925)

8 3 and 7 (5236)

9 mortality/ (537684)

10 mortalit*.mp. (919964)

11 9 or 10 (919964)

12 8 and 11 (1082)

Global Health

Database: Global Health <1973 to 2014 Week 46>

Search Strategy:

--------------------------------------------------------------------------------

1 "Treatment outcome of new smear positive pulmonary tuberculosis patients in Penang, Malaysia".fc_titl. (1)

2 "Predictive factors for mortality among non-HIV-infected patients with pulmonary tuberculosis and respiratory failure".fc_titl. (1)

3 "Hospitalizations for Tuberculosis in the United States in 2000".fc_titl. (0)

4 "Initial presentations predict mortality in pulmonary tuberculosis patients".fc_titl. (1)

5 "Risk factors related with mortality in patient with pulmonary tuberculosis".fc_titl. (0)

6 "Factors associated with mortality in hospitalized patients with newly diagnosed tuberculosis".fc_titl. (1)

7 "High mortality in adults hospitalized for active tuberculosis in a low HIV prevalence setting".fc_titl. (1)

8 "Mortality of Patients Hospitalized for Active Tuberculosis in Israel".fc_titl. (0)

9 "Prognostic factors in tuberculosis related mortalities in hospitalized patients".fc_titl. (0)

10 "Factors associated with mortality in tuberculosis patients".fc_titl. (0)

11 or/1-10 (5)

12 exp tuberculosis/ (33746)

13 tubercul*.mp. [mp=abstract, title, original title, broad terms, heading words, identifiers, cabicodes] (44028)

14 12 or 13 (44028)

15 hospital admission/ or hospital care/ or hospital stay/ (5944)

16 hospitalized.mp. (18553)

17 hospitalization.mp. (14788)

18 in-hospital.mp. (9822)

19 or/15-18 (40604)

20 14 and 19 (1212)

21 hospital*.mp. (170115)

22 19 or 21 (170115)

23 14 and 22 (6555)

24 mortality/ (61921)

25 mortalit*.mp. [mp=abstract, title, original title, broad terms, heading words, identifiers, cabicodes] (113527)

26 24 or 25 (113527)

27 20 and 26 (340)

28 23 and 26 (1029)
